# Genomic, epigenomic, and transcriptomic signatures of prostate cancer between African American and European American patients

**DOI:** 10.3389/fonc.2023.1079037

**Published:** 2023-02-28

**Authors:** Claire Stevens, Alexandria Hightower, Sarah G. Buxbaum, Sara M. Falzarano, Suhn K. Rhie

**Affiliations:** ^1^ Department of Biochemistry and Molecular Medicine, USC Norris Comprehensive Cancer Center, Keck School of Medicine of USC, Los Angeles, CA, United States; ^2^ CaRE2 Program, Florida-California Health Equity Center, Los Angeles, CA, United States; ^3^ Department of Epidemiology and Biostatistics, College of Pharmacy and Pharmaceutical Sciences, Institute of Public Health, Florida A&M University, Tallahassee, FL, United States; ^4^ Department of Pathology, Immunology, and Laboratory Medicine, University of Florida College of Medicine, Gainesville, FL, United States

**Keywords:** prostate cancer, racial disparity, African American (AA), European American (EA), genomics, epigenomics, transcriptomics

## Abstract

Prostate cancer is the second most common cancer in men in the United States, and racial disparities are greatly observed in the disease. Specifically, African American (AA) patients have 60% higher incidence and mortality rates, in addition to higher grade and stage prostate tumors, than European American (EA) patients. In order to narrow the gap between clinical outcomes for these two populations, genetic and molecular signatures contributing to this disparity have been characterized. Over the past decade, profiles of prostate tumor samples from different ethnic groups have been developed using molecular and functional assays coupled with next generation sequencing or microarrays. Comparative genome-wide analyses of genomic, epigenomic, and transcriptomic profiles from prostate tumor samples have uncovered potential race-specific mutations, copy number alterations, DNA methylation, and gene expression patterns. In this study, we reviewed over 20 published studies that examined the aforementioned molecular contributions to racial disparities in AA and EA prostate cancer patients. The reviewed genomic studies revealed mutations, deletions, amplifications, duplications, or fusion genes differentially enriched in AA patients relative to EA patients. Commonly reported genomic alterations included mutations or copy number alterations of *FOXA1, KMT2D, SPOP, MYC*, *PTEN*, *TP53*, *ZFHX3*, and the *TMPRSS2*-*ERG* fusion. The reviewed epigenomic studies identified that CpG sites near the promoters of *PMEPA1*, *RARB*, *SNRPN*, and *TIMP3* genes were differentially methylated between AA and EA patients. Lastly, the reviewed transcriptomic studies identified genes (e.g. *CCL4, CHRM3*, *CRYBB2*, *CXCR4, GALR1*, *GSTM3*, *SPINK1*) and signaling pathways dysregulated between AA and EA patients. The most frequently found dysregulated pathways were involved in immune and inflammatory responses and neuroactive ligand signaling. Overall, we observed that the genomic, epigenomic, and transcriptomic alterations evaluated between AA and EA prostate cancer patients varied between studies, highlighting the impact of using different methods and sample sizes. The reported genomic, epigenomic, and transcriptomic alterations do not only uncover molecular mechanisms of tumorigenesis but also provide researchers and clinicians valuable resources to identify novel biomarkers and treatment modalities to improve the disparity of clinical outcomes between AA and EA patients.

## 1 Introduction

Prostate cancer is the second most common cancer in men with one of the highest incidence rates in the United States (1). It has the second highest mortality rate relative to other malignancies, but the severity of clinical outcomes is reported to vary by race and ethnicity (1). Specifically, African American (AA) men have the highest prostate cancer incidence rate amongst racial or ethnic groups in the United States (2). For example, 1 in 6 AA men are diagnosed with prostate cancer in their lifetime, compared to 1 in 8 European American (EA) men, and the incidence is nearly 60% higher for AA men (2). Moreover, AA prostate cancer patients present with higher grade and stage tumors and have a nearly 2-fold higher mortality rate when compared to EA prostate cancer patients (2). Specifically, AA men have a prostate cancer mortality rate of 37.4 per 100,000 in the period of 2014-2018 versus 19.3 per 100,000 among white non-Hispanic men (3). Furthermore, prostate cancer has a higher growth and metastatic transformation rate for AA men compared to EA men (4). It has been reported that low-grade prostate cancer cells grow and spread more quickly in AA than men of other races (5).

This discrepancy between clinical outcomes appears to be attributable to socioeconomic factors that may cause barriers to medical access, diagnosis, and treatment among AA men (6). For example, AA men experience substandard testing of prostate specific antigen (PSA) relative to their EA counterparts, leading to limited access to early detection of prostate cancer (7–9). In addition to socioeconomic factors, biological factors may further widen the gap between clinical outcomes for AA men relative to EA counterparts. For example, Cheng et al. reported that AA men were more likely to develop prostate cancer relative to their EA counterparts in California after correcting for socioeconomic status (10). Another study emphasizes that the investigation of race-specific biological differences needs to be viewed through a multifactorial lens because factors such as environment, social status, and genetic inheritance can lead to different mechanisms of prostate tumorigenesis in a combinatorial manner (11). Currently, there is lack of comprehensive understanding on the different biological contributions to prostate tumorigenesis between AA and EA patients.

To elucidate possible biological determinants of racial disparities in prostate cancer, prostate tumor cells and tissues from AA and EA patients were obtained through various methods (e.g. transrectal ultrasound (TRUS)-guided biopsy, transperineal biopsy, transurethral resection, prostatectomy, radical prostatectomy), and genetic and molecular assays have been performed with the obtained samples. Over the past two decades after next generation sequencing (NGS) technologies were introduced, prostate tumor samples have been studied using genetic and molecular assays that are coupled with sequencing. Using NGS and microarray techniques that can evaluate genome-wide signals at once, many studies have characterized genetic (e.g. mutations, copy number alterations, fusions) and molecular features (e.g. epigenetic alterations, gene expression changes) of prostate tumors through the lens of AA and EA patient outcome disparities.

**Figure 1:**
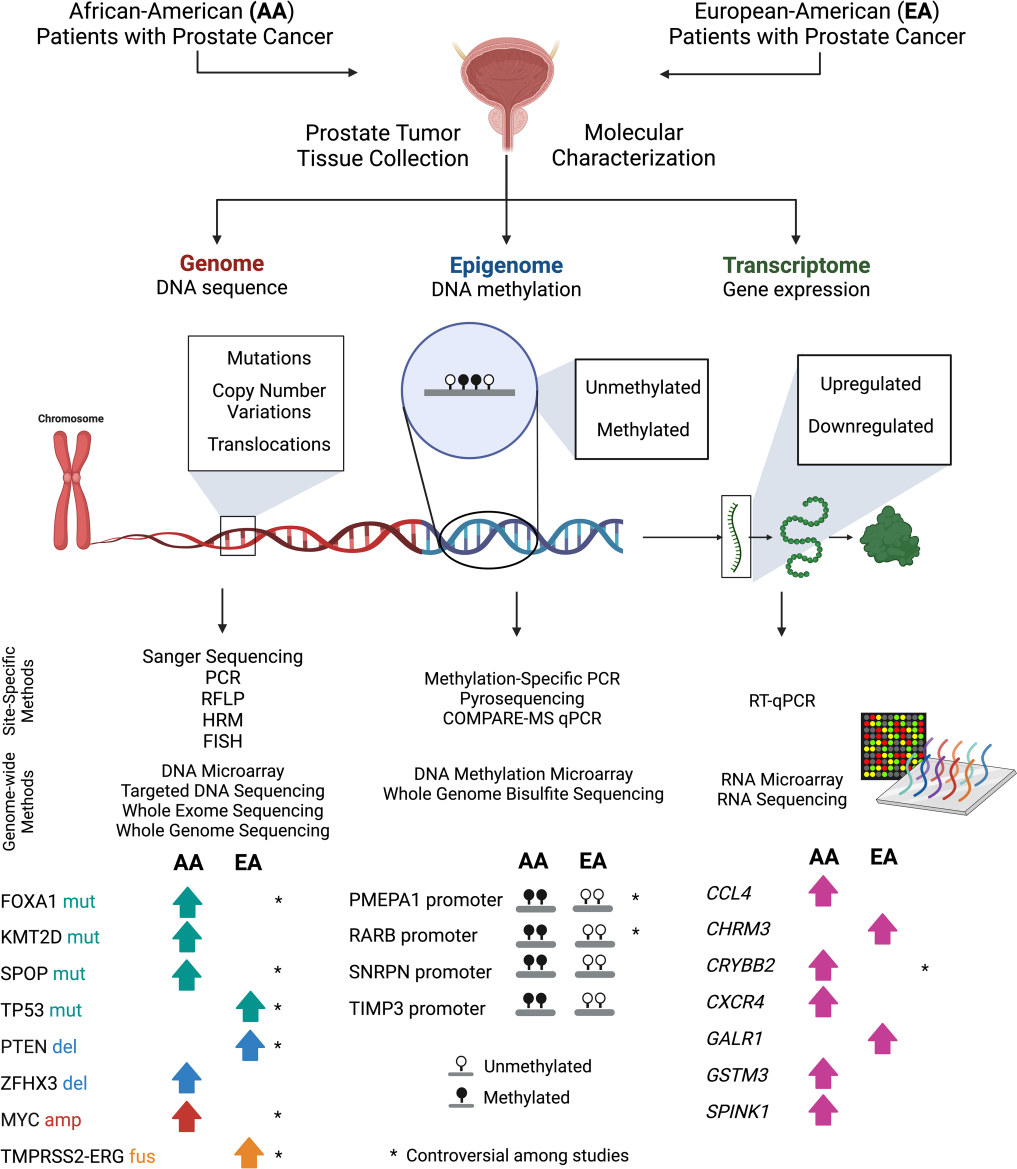
Approaches to compare molecular genetic signatures in prostate tumor tissue samples obtained from AA and EA patients. In reviewed studies, prostate tumor tissue samples from both EA and AA patients were collected by either biopsy or prostatectomy. DNA or RNA material was extracted from aforementioned samples and then prepared for molecular characterization at the genome, epigenome, and transcriptome levels. Studies profiled the signatures of prostate tumor tissues using site-specific techniques and genome-wide utilized high-throughput hybridization (microarray) and next-generation sequencing (NGS) technologies. Subsequently, AA and EA patient sample data was compared to uncover potential molecular differences between the two subpopulations. Key molecular differences found by several studies and discussed in this review were included. Comparisons that were not found to have consistent data across studies (or controversial) was denoted with an asterisk (*). PCR, Polymerase chain reaction; RFLP, Restriction fragment length polymorphisms; HRM, High resolution melting analysis; FISH, Fluorescence *in situ* hybridization; and COMPARE-MS, Combination of methylated-DNA precipitation and methylation-sensitive restriction enzymes assay.

Here, we have listed findings from over 20 published studies that profiled the genomes, epigenomes, and transcriptomes of prostate tumor tissues of AA and EA patients. Studies were identified by inputting the following key words into the PubMed search query: “African-American” AND “prostate cancer”. For each category (genome, epigenome, transcriptome), search terms were changed to DNA, methylation, and RNA, respectively. Studies were deemed to be within the scope of this review if they were written in the English language and performed using prostate tumor tissue samples from AA and EA patients, clearly reporting the cohort sizes and methods used. Moreover, we only included studies that directly compared genetic, epigenetic, or transcriptomic profiles of prostate tumor tissues between AA and EA patients (**Tables 1**–**3**). These studies uncovered potential race-specific mutations, copy number alterations, fusions, and aberrant DNA methylation and gene expression patterns, using varied methods of DNA, DNA methylation, and RNA analysis, respectively (**Figure 1**). This review, which details a bigger picture of prostate cancer biological signatures, will provide clinicians and researchers with a better understanding of molecular mechanisms of prostate tumorigenesis and facilitate the development of potential biomarkers and treatment modalities to narrow the gap between AA and EA patient outcomes.

**Table 1:** Studies that compared genomic features between AA and EA patients.

PubMed ID	Name	Sample	Total Cohort Size	Cohort Details	Method	Genes Examined	Key Findings
25056375	Khani F et al, 2014 (12)	Radical prostatectomy tissue	218	105 AA and 113 EA	HRM followed by Sanger sequencing, FISH	FISH: *ERG, PTEN* Sequencing: *SPOP*	Less *ERG* rearrangements in AA Less *PTEN* deleted in AA Less *SPOP* mutated in AA
32651179	Koga et al., 2020 (13)	FFPE tissue	861	WES: 250 AA and 611 EA Targeting sequencing: 436 AA and 3018 EA Microarrays: 171 AA and 626 EA	WES, Hybridization capture-based targeted DNA sequencing, SNP 6.0 microarrays	*CDK12, FOXA1, AR, BRAF, BRCA2, BRIP1, CDKN1B, CDK6, CTNNB1, ERF, ETV3, FGFR1, FLCN, JAK1, KEL, KMT2D, KDM6A, MAP3K1, MCL1, MED12, MYC, NOTCH2, PIK3CA, PTCH1, PTEN, SPOP, TMPRSS2, TP53, CCND1, KMT2D, ZFHX3, NKX3-1*	Less *ERG* rearrangements in AA More *KMT2D, ERF, SPOP* loss of function mutations in AA More *MYC, CCND1, HGF* amplifications in AA More *ZFHX3, ETV3* deleted in AA Less *PTEN* deleted in AA
24948877	Koochekpour et al., 2014 (14)	Radical prostatectomy tissue	300	200 AA and 100 EA	Pyrosequencing	*AR*	More *AR* mutated in AA
26921337	Lindquist et al., 2016 (15)	Radical prostatectomy tissue	24	24 AA and publicly available data sets (TCGA and COSMIC) for EA*	WGS	*TMPRSS2-ERG, PTEN, CDC27-OAT*	More *CDC27-OAT* rearranged in AA More *FOXA1* mutated in AA Less *TP53* mutated in AA Less *PTEN* deleted in AA Less *TMPRSS2-ERG* rearranged in AA Less *MYC* amplifications in AA
33115829	Liu et al., 2020 (16)	FFPE tissue	1031	171 AA and 860 EA	Exome sequencing for 39 selected genes OncoScan CNV microarrays	*AKT1, APC, AR, ATM, BRAF, BRCA2, CASZ1, CBX7, CDK12, CDKN1B, CHD1, CST2, CTNNB1, FOXA1, FRG1, HRAS, IDH1, IL6ST, KDM6A, KIF5A, KMT2C, KMT2D, MED12, NIPA2, NKX3-1, PIK3CA, PIK3CB, PTEN, RB1, REST, SCN11A, SPOP, TBL1XR1, THSD7B, TP53, ZFHX3, ZMYM3, ZNF595, ZNF770*	More *ZMYM3, FOXA1, APC, ATM, BRCA2, KDM6A, KMT2C, KMT2D, MED12, ZFHX3, MAP3K7, BNIP3L* mutated in AA Less *SPOP* and *TP53* mutated in AA More *CNAs in MYC, THADA, NEIL3, LRP1B, BUB1B, MAP3K7, BNIP3L*, and *RBI* in AA Less deleted of *RYBP, TP53*, and *TMPRSS2-ERG* in AA
32168400	Liu et al., 2020 (17)	FFPE tissue	288	147 AA and 141 EA	PCR-RFLPs	*TP53*	No difference in *TP53* mutation frequency between EA and AA

*Generated as part of a previous study.

**Table 2:** Studies that compared DNA methylation features between AA and EA patients.

PubMed ID	Name	Sample	Total Cohort Size	Cohort Details	Method	Genes Examined	Key Findings
33374332	Barry et al., 2020 (18)	FFPE tissue	89	43 AA and 46 EA	Pyrosequencing	*MYC*	Strong association for *MYC* DNA methylation at one CpG site, but no CpG locations studied were observed to be significantly differentially methylated
25864488	Devaney et al., 2015 (19)	Radical prostatectomy tissue	6	3 AA and 3 EA	Human Methylation450 BeadChip arrays and pyrosequencing	*ABCG5, ACOT7, MST1R, SPTB, SHANK2, SNRPN, WDR70*	Hypermethylation of *SNRPN, MST1R, ABCG5* in AA relative to EA
15800905	Enokida et al., 2005 (20)	Radical prostatectomy tissue	121	44 AA and 77 EA	Methylation specific PCR	*GSTP1*	No significant differences between races
20606036	Kwabi-Addo et al., 2010 (21)	Radical prostatectomy tissue	100*	39 AA and 67 EA*	Methylation specific PCR	*GSTP1, AR, RARB, SPARC, TIMP3, NKX2-5*	Differential methylation of *RARB, SPARC, TIMP3*, and *NKX2-5* between AA and EA patients No significant differences in methylation of *GSTP1* between AA and EA patients
26902887	Rubicz et al., 2019 (22)	FFPE tissue	76	76 AA and 476 EA**	Human Methylation450 BeadChip arrays	450,000 CpG sites throughout the genome	Hypermethylation of *STOX7, SNRPN, TIMP3*, and *PMEPA1* in AA relative to EA with no corresponding changes in mRNA levels
24694733	Sharad et al, 2014 (23)	Radical prostatectomy tissue	77	35 AA and 42 EA	COMPARE-MS (methylated-DNA precipitation and methylation specific restriction enzymes) followed by qPCR	*PMEPA1, GSTP1*	Hypermethylation of *PMEPA1* in AA relative to EA No significant difference in hypermethylation of *GSTP1* between AA and EA
12692786	Woodson et al., 2004 (24)	FFPE tissue	111	47 AA and 67 EA	Methylation specific PCR	*GSTP1, CD44, E-cadherin*	No significant difference in hypermethylation of *GSTP1* between AA and EA Hypermethylation of *CD44*, higher frequency amongst AA patients relative to EA patients No differential methylation of *E-cadherin*

*Based on Table 1 of Kwabi-Addo et al. (21)

**Generated as part of a previous study.

FFPE, Formalin-fixed, paraffin-embedded.

**Table 3:** Studies that compared transcriptomic features between AA and EA patients.

PubMed ID	Name	Sample	Total Cohort Size	Cohort Details	Method	Key Findings
31107158	Echevarria et al., 2019 (25)	Radical prostatectomy tissue	635	127 AA 508 EA	Human Exon 1.0 ST microarrays	*APOD, BCL6, EMP1, MYADM, SRGN* and *TIMP3* upregulated in AA
27359067	Hardiman et al., 2016 (26)	Radical Prostatectomy issue	27	10 AA and 17 EA	TruSeq RNA library preparation kit	Immune and inflammatory genes (ie. *IL2RG, CD1C, CD207, CCL4, CCL8, CXCR4*) upregulated in AA
34680291	Hardiman et al., 2021 (27)	Prostatectomy and biopsy tissue	60	33 AA and 27 EA	TruSeq RNA library preparation kit	*THBS4, CREB3L1, TNN, COL4A4, COL4A3, COL2A1, FGF12, MYC, GNG13, AGTR1, F2RL2, NPY4R*, and *GRIN3A* upregulated in AA* SGK1, ANGPT, FGF11, IL4, IL6, ANGPT4, THBS2, FLT4, NTRK2, PIK3R6, LAMA5 MET, GABRP, ADORA2B, TACR1, TAAR1* and *GABRQ* downregulated in AA
34692584	Nagaya et al., 2021 (28)	Radical prostatectomy tissue	61	31 AA and 30 EA	Ovation universal RNA-seq library preparation kit	*S1PR3* upregulated in AA and *GALR1, CHRM3* and *NPFFR1* downregulated in AA
34316327	Rahmatpanah et al., 2021 (29)	Radical prostatectomy tissue	45	15 AA and 30 EA	TruSeq RNA library preparation kit	*GRIN3A* downregulated in AA
34083737	Rayford et al., 2021 (30)	Radical prostatectomy tissue	1,152	596 AA and 556 EA	Human Exon 1.0 ST microarrays	*CRYBB2* and *GSTM3* upregulated in AA
19724911	Timofeeva et al., 2009 (31)	Radical prostatectomy tissue	27	14 AA and 13 EA	GeneChip HG-U133A 2.0 microarrays, qRT-PCR	*SOS1* upregulated in AA
18245496	Wallace et al., 2008 (32)	Radical prostatectomy tissue	69	33 AA and 36 EA	GeneChip HG-U133A 2.0 microarrays	No difference observed

## 2 Genomic alterations linked to African American prostate cancer patients

### 2.1 Methods to profile genomic alterations in cancer

Cancers develop due to the accumulation of genetic alterations such as mutations, amplifications, deletions, and fusions. Deletions and inactivating (loss-of-function) mutations are often found at tumor suppressor genes to dysregulate cell division. Conversely, amplifications and activating mutations are found at oncogenes to increase cancer cell proliferation and survival. Moreover, genetic alterations are observed at non-coding regions such as regulatory regions to activate or inactivate genes involved in carcinogenesis.

To identify genetic alterations in cancer cells, polymerase chain reaction (PCR)-based assays, DNA sequencing and hybridization technology have been applied. Classical techniques that have been utilized to parse out short sequences include Sanger sequencing, pyrosequencing, PCR-restriction fragment length polymorphisms (PCR-RFLP) analysis, and High-Resolution Melting (HRM) analysis (33–37). An example of a classical hybridization technique is Fluorescence *in situ* hybridization (FISH), which utilizes a fluorescently labeled probe targeting a specific sequence to detect copy number variations (CNV). The major drawback of all of the above-mentioned methods is that they are limited to specific regions within the genome. To find high-throughput genetic alterations across the genome, DNA microarray-based and NGS techniques were developed (38, 39). DNA microarray technology determines the number of copies of particular genomic regions through sequence-primer hybridization (40, 41). CNV microarrays such as OncoScan can characterize CNVs in 900 cancer genes and 300kb region outside of the cancer genes throughout the genome, based on hybridization (42).

Two main NGS applications have allowed for high-throughput detection of genetic alterations in tumors: targeted DNA sequencing, and whole genome sequencing (WGS) (43, 44). While WGS determines the sequence of the entire genome, targeted DNA sequencing methods such as hybridization capture NGS and amplicon-based NGS sequence specific DNA fragments (44, 45). One major application for targeted DNA-sequencing is the targeting of exons throughout the genome, also known as whole exome sequencing (WES). Overall, DNA microarrays, WES, and WGS have been essential to elucidating cancer-associated mutations and focal copy number alterations, and large-scale chromosomal copy number alterations.

### 2.2 Genomic alterations frequently observed in prostate tumors

Prostate tumors often harbor inactivating, or loss of function (LOF), mutations or deletions in tumor suppressor genes involved in pathways regulating cell cycle, DNA repair, and transcriptional regulation. Genetic alterations identified at the greatest frequency in prostate cancer related to cell cycle include mutations at *TP53, RB1*, *PTEN*, and deletions at chromosome 10q containing *PTEN* (46–48). Several studies state that *ZFHX3*, which functions to inhibit prostate carcinogenesis by suppressing *MYC* overexpression, is correlated to improved patient survival (49). It is reported that *ZFHX3* is deleted or contains a LOF mutation in prostate tumors (50). Notable tumor suppressor genes involved in transcriptional regulation that are frequently deleted or mutated in prostate tumors include *SPOP, MED12, CHD1*, and *ZNF292* (51).

The aforementioned genetic alterations are somatically acquired during tumorigenesis. Germline mutations including *BRCA1*, *BRCA2*, *HOXB13*, *CHEK2*, and *ATM* mutations have been associated with hereditary prostate cancer (52). One of the most common mutations is in *BRCA2* (47, 52). *BRCA1/2*, known as tumor suppressor genes, mediate double-strand break DNA repair (53). *BRCA1/2* mutations are not only associated with an increased risk of prostate cancer, but also with an aggressive prostate cancer phenotype, such as higher grade, advantaged stage, and poor survival (54, 55). *HOXB13* is important in prostate development and this mutation was observed in 0.7-1.4% of prostate cancers, and 6% of early-onset prostate cancer (56). *HOXB13* mutations are also associated with an increased hereditary prostate cancer risk (57, 58). Lu et al. found that the *HOXB13* mutation disrupts the interaction between HOXB13 and histone deacetylase 3 (HDAC3), an epigenetic modifier (59).

Genetic alterations such as fusions, amplifications, translocations, and gain-of function (GOF) mutations are found in oncogenes in prostate cancer. As reported in various studies, androgen receptor (*AR*) has been increasingly implicated in prostate cancer (60). AR is a key transcription factor playing a critical role in prostate cancer initiation and progression. It leads to prostate cancer cell proliferation by mediating transcription of pro-mitotic genes (61, 62). Androgen binding induces a conformational change, resulting in its nuclear translocation (63, 64). Subsequently, AR binds at specific genomic regions and activates transcription of its numerous target genes (64).

Since up to 90% of prostate cancer is dependent on androgens at diagnosis, androgen signaling has been considered a pivotal therapeutic target. Androgen deprivation therapy (ADT), either alone or in combination with chemotherapy, is the mainstay of initial treatment for advanced, high-grade and stage, prostate cancer, albeit many patients eventually will develop progressive disease referred to as castration-resistant prostate cancer (CRPC) (64–66). *AR* genetic alterations including mutations and amplifications are observed in prostate cancer as well as alternative splicing events. There are four domains of AR: amino-terminal transcriptional domain (NTD), DNA-binding domain (DBD), hinge region, and a carboxy-terminal ligand-binding domain (LBD) (66). Splicing variants encode truncated AR forms, such as AR-V7, lacking the LBD and thus making the protein constitutively active regardless of the presence of androgens (67). Interestingly, *AR* mutations and amplification events are nearly exclusively found in metastatic prostate tumor samples, but not in primary tumor samples. *AR* mutation burden usually increases with tumor stage, and alterations oftentimes involve missense mutations in the LBD (68). In this way, there is less specificity of the domain overall, as AR activation can be induced by various ligands beyond its non-pathological activators (68, 69).

Downstream and upstream effectors of AR are also found to be altered in primary tumor samples (47). The most frequent gene rearrangements found in primary prostate tumors are those involving *TMPRSS2* (Transmembrane Serine Protease 2) and members of the *ETS* (erythroblast transformation specific transcription factor) family transcription factors. The *ETS* family includes *ERG*, *ETV1*, *ETV4*, and *FLI1* which can all form fusions with *TMPRSS2*. In the Cancer Genome Atlas (TCGA) prostate adenocarcinoma data, 53% of tumors were found to have *ETS* family gene fusions. The *ETS* family gene members involved in the fusions, the most common being *ERG* (46%), were found to be mutually exclusive. The promoter of *TMPRSS2-ERG* gene is reported to be bound by AR. Interestingly, *AR* genetic aberrations and the fusion proteins under its regulation are seen at variable relative levels. Moreover, most of the *TMPRSS2-ETS* fusion positive tumors also contained *PTEN* deletions (47).

The next most common genetic alterations found in prostate tumors include mutations in *SPOP, FOXA1*, and *IDH1*. Interestingly, *SPOP* and *FOXA1* mutant tumors were found to be both mutually exclusive with *TMPRSS2-ETS* fusions and had higher AR transcriptional activity (47). Lastly, the presence of amplifications, insertions, and deletions, also known as copy number alterations (CNAs), have been shown to be directly correlated to disease severity. For example, *CHD1* deletions and *SPOP* mutations frequently co-occur in prostate tumors (47). Prostate tumors with whole chromosomal arm gains or losses are associated with high grade, Gleason score, and PSA levels (47). Common amplifications span the oncogenes *MYC* (8q24.21), *CCND1* (11q13.2), *FCFR* (12p11.21), and *NSD3* (8p11.23) and common deletions span tumor suppressor genes *PTEN* (10q23)*, TP53* (17p13.1)*, CDKN1B* (12p13.1)*, MAP3K1* (5q11.2 & 6q.12-22)*, FANCD2* (3p26)*, SPOPL* (2q22.1), and *FOXP1/RYBP/SHQ1* (3p13) (47). Interestingly, aggressive prostate tumors with greater mutational burden or with higher CNA frequencies have more mutations at *KMT2D, TP53*, and *KDM6A* and a higher frequency of *MYC* amplifications (47).

### 2.3 Genomic alterations differentially identified in AA and EA prostate cancer patients

Due to the reported outcome disparities between AA and EA men with prostate cancer, many studies have sought to evaluate potential genetic alteration differences between tumors obtained from these two subgroups. When we searched for studies that performed their own DNA-sequencing (WGS and WES) or genetic alteration analysis in prostate tumor tissue samples from AA and EA, we found six studies that fulfilled inclusion criteria (12–17) (**Table 1**). All analyzed data were based on self-identified race, in lieu of comparing samples using ancestry-specific analysis. Sample sizes for participated AA and EA prostate cancer patients varied across studies, as well as methodologies.

Among somatic mutations commonly found in prostate cancer, *TP53, PTEN*, and *ZFHX3* mutations affect cell cycle and growth. As previously mentioned, *TP53* loss of function mutations are relatively common in prostate tumor samples. Two studies, Lindquist et al. and Liu et al., which evaluated DNA with WGS and targeted exome sequencing, respectively, reported that AA patients had relatively less *TP53* inactivating mutations than EA patients (15, 16). Another two studies, Koga et al. and Liu et al., which used WES and PCR, respectively, found that there was no significant difference in *TP53* mutation frequency between AA and EA (13, 17). The correlation between *PTEN* loss and race is largely unclear. The frequency of *PTEN* loss was not found to be significantly different between AA or EA prostate cancer patients by Khani et al. (12) and and Liu et al. (16); the two studies used FISH and the Affymetrix OncoScan FFPE SNP CNV microarray to assess CNV status, respectively (12, 16). However, two other studies reported that *PTEN* loss was less frequent in AA patient tumors using WGS and WES, respectively (13, 15). The *ZFHX3* gene has been found to be more frequently deleted or contain a LOF mutation in AA patients relative to EA across several studies (13, 16). This distinction can prove important to the treatment of AA prostate cancer since functional *ZFHX3* is necessary for effective ESR2 (aka ERß) agonist treatment (49).

Genetic alteration frequency of two genes, *SPOP* and *KMT2D*, which regulate the transcriptional process were investigated between AA and EA across multiple studies. Results describing the frequency of *SPOP* mutations in both AA and EA tumor samples varied. For example, Koga et al. (13) found a higher frequency of *SPOP* mutations in AA compared to EA when considering all tumors, regardless of primary or metastatic, whereas Liu et al. (16) found that the frequency of *SPOP* mutations was lower in AA patients. Additionally, two studies found no differences in mutation frequency between AA and EA (12, 15). *KMT2D* mutations, which are associated with more aggressive disease, were found at a greater frequency in AA patients in multiple studies (13, 16).

Genetic alterations in genes related to androgen signaling (*AR* and *FOXA1*) were also evaluated by multiple studies. The study by Koochekpour et al, which used PCR analysis, found that *AR* mutations were more frequent in AA prostate cancer patient samples than EA (14), but two other studies found no difference in mutation frequency (13, 16). *FOXA1* was found to have a greater mutation frequency in AA patients (15), whereas another study found it not to be statistically significantly different (13). *MYC* amplification was a point of contention between studies. Lindquist et al. reported that AA patients were less likely to have *MYC* amplification (15), whereas other studies reported that AA patients were more likely to have *MYC* amplification (13, 16).

Fusion events were found in many prostate tumors, but these findings were observed in datasets overwhelmingly obtained from EA patient populations, such as the TCGA dataset. Multiple studies found that *TMPRSS2-ERG* fusions were less prevalent in AA patients relative to EA patients (13, 15, 16, 70, 71). Although the absence of these fusions has not been associated with a worse prognosis, the significant difference in prevalence demonstrates the need to evaluate previously established genetic biomarkers of disease in AA patient populations (72). Interestingly, a novel gene fusion, *CDC27-OAT*, was shown to be either specific or more common in AA patients (15).

Other genetic alterations not previously associated with prostate cancer were found to be significantly different between AA that EA patient samples. Lindquist et al. described that those frequent mutations in two genes (*MUC3A* and *PRIM2*) were found more commonly in AA tumor samples, which could be potentially carcinogenic (15). These genes encode proteins involved in cell growth and survival, and DNA replication, respectively. The study by Koga et al. identified a novel deletion spanning *ETV3* (1q23.1), another *ETS* transcription factor, in AA tumor samples (13).

Overall, most of these studies addressing racial differences did not have consistent results for genes related to prostate carcinogenesis although *ZFHX3* and *KMT2D* alterations were found in multiple studies at a greater frequency in AA patients and were associated with increased disease severity. It is possible that a subset of genetic alterations may at least in part contribute to the clinical disparities seen between AA and EA prostate cancer patients. It is therefore important to fully interrogate more samples to understand the contribution of genomic difference to the severity of disease using DNA-sequencing techniques.

## 3 Epigenomic alterations linked to African American prostate cancer patients

### 3.1 Methods to profile epigenomic alterations in cancer

Epigenetic alterations change the chromatin state and structure to regulate gene expression without changes in DNA sequence in cancer cells. Chromatin state and structure are important for the maintenance of cell states. Nucleosome positioning, histone modifications as well as DNA methylation define the status and identity of each cell, and they are maintained throughout the cell cycle (73–77). Non-coding RNAs (ncRNAs) are also reported to regulate gene expression and chromatin state to control cell proliferation and differentiation (78). Among those epigenetic alterations, DNA methylation analysis is used most often because DNA samples obtained for the DNA methylation analysis are easily isolated and stored from diverse tissue types. Additionally, DNA methylation analysis can be performed with a small amount of DNA (79).

Classical DNA methylation methodologies include methylation-specific PCR (MSP), pyrosequencing, and Luminometric Methylation Assay (LUMA). MSP involves PCR at a specific CpG site of-interest using site-specific primers, one for methylated CpG and the other for unmethylated CpG detection (80). Although this method interrogates the DNA methylation status at a specific site of interest, the drawback is that only one or two CpG sites can be assessed at a time. Additionally, MSP is difficult to perform in regions that are not CpG islands (81). Pyrosequencing detects DNA methylation levels of CpG sites in a PCR product (82). The advantages of this method are that it is time-efficient, quantitative, and can detect even small differences in methylation (83). However, primer design is difficult, and only a short region can be analyzed (83).

To profile DNA methylation at multiple sites at once, multiple techniques were developed. LUMA technology, which was based on the combined methylation-sensitive restriction enzyme DNA cleavage and pyrosequencing-based polymerase extension, was one of the earliest developed methods (84). A combined method such as Combination of methylated-DNA precipitation and methylation-sensitive restriction enzymes (COMPARE-MS) assay, which uses PCR products of DNA first digested by methylation-sensitive restriction enzymes then precipitated by methyl-binding domain polypeptides, was also developed to detect CpG island DNA methylation (85). However, these assays are only capable of detecting differences in DNA methylation within methylation-sensitive restriction enzyme cut sites, which are not uniformly distributed in the genome. Therefore, these assays cannot exhaust all of the CpG sites in the genome.

To better profile DNA methylation sites, genome-wide, high-throughput techniques coupled with NGS and microarrays have been developed. Methyl-CpG-binding domain sequencing (MBD-seq) and methylated DNA immunoprecipitation sequencing (MeDIP-seq) are based on affinity purification using antibodies (86). Genomic DNA is prepared, sheared, denatured, and then immunoprecipitated. Pull down of methylated DNA is possible by using MBD-seq or MeDIP-seq methodologies (81). MBD proteins bind to double-strand methylated DNA using the methyl-binding domain while MeDIP uses a 5-methylcytosine monoclonal antibody against single-strand DNA (87). Both techniques are cost-effective, are capable of distinguishing between 5mC and 5hmC, induce no mutations, and provide no limitations to enzyme recognition sites. However, both methods are biased toward hypermethylated regions (86). MBD methods tend to be more sensitive to enrichment of CpG islands compared to MeDIP, while MeDIP provides relatively superior profiling of enrichment regions with lower CpG density (86).

Subsequent technologies using bisulfite conversion coupled with hybridization (i.e. DNA methylation microarrays) or sequencing (i.e. whole genome bisulfite sequencing) have been developed to provide insight as to the DNA methylation state of regions of-interest simultaneously and globally. The DNA methylation arrays that are most used were developed by Illumina. The Illumina methylation assay uses BeadChip to generate a genome-wide methylation profile. Similar to pyrosequencing, this method quantifies methylation levels at individual CpG loci within the genome (88). The bisulfite-converted DNA is amplified, fragmented, and hybridized to probes on the microarray, providing targeted-enrichment of methylated regions (89). The main advantages of the DNA methylation microarray method include cost-effectiveness, time-efficiency, and the low DNA input required (51, 89, 90). The initial Infinium Human Methylation27 (HM27) BeadChip contains 27,578 CpG sites. Later, Illumina developed the Infinium Human Methylation450 (HM450) BeadChip assay which interrogates 482,421 CpG sites, including 90% of the sites on the HM27 (89). More recently, the Infinium Methylation EPIC (EPIC) BeadChip has been developed, containing over 850,000 CpG sites including promoters, enhancers, and open chromatin regions (91).

Whole genome methylation sequencing utilizes NGS techniques to obtain DNA methylation data that can include all CpG sites within the genome at single nucleotide resolution (76). Whole genome bisulfite sequencing (WGBS) allows the identification of methylation state at almost every CpG site in the genome, providing highly integrated single base resolution DNA methylation patterning (92). However, this method is high cost, unable to distinguish between 5mC and 5-Hydroxymethylcytosine (5hmC) and causes substantial DNA degradation after bisulfite treatment (86). Reduced representation bisulfite sequencing (RRBS) applies WGBS techniques to specific regions of interest. Because only a fraction of the genome is sequenced, RRBS is highly sensitive and cost-effective compared to WGBS (86).

DNA methylation changes reported by these techniques in cancer results in the silencing of tumor suppressor genes or activation of oncogenes. Compared to normal cells, the promoter regions of tumor suppressors are hypermethylated whereas the promoter regions of oncogenes are hypomethylated (93). Moreover, cancer-specific enhancer regions are hypomethylated (94, 95). Furthermore, long-range hypomethylated regions such as partially methylated domains are detected in cancer cells (96). The hypomethylation and hypermethylation of specific CpG sites in cancer allows for the understanding of molecular mechanisms of dysregulated of tumor suppressors and oncogenes (97).

### 3.2 Heterogeneous DNA methylation patterns among prostate tumors

To understand and characterize DNA methylation states of prostate tumor samples relative to normal prostate tissue, several DNA methylation studies have been performed (47, 92, 98–125). For example, in a review written by Lam et al. (99), six genes (*GSTP1*, *APC*, *RARB*, *PITX2*, *CCND2*, and *PTGS2*) along with their corresponding CpG sites were identified as having prognostic importance across several prostate cancer studies (99–125). Of the six genes, *GSTP1, APC, RARB, CCND2*, and *PTGS2* were tumor suppressors identified as having been hypermethylated in prostate tumor tissues. *PITX2*, an oncogene that also bears importance in lung adenocarcinoma, was identified as being hypomethylated in prostate cancer.

Moreover, TCGA, which profiled DNA methylation levels of over 300 prostate tumor tissues using Illumina HM450 arrays, revealed heterogeneous DNA methylation patterns amongst 8 prostate tumor molecular subtypes including: *TMPRSS2-ERG* fusion, *TMPRSS2-ETV1* fusion, *TMPRSS2-ETV4* fusion, *TMPRSS2-FLI1* fusion, *SPOP* mutation, *FOXA1* mutation, *IDH1* mutation, and tumors without genetic alterations (47). For example, *IDH1* mutant tumors exhibited heavy hypermethylation throughout the genome. *SPOP* and *FOXA1* mutant tumors had similar DNA hypermethylation patterns with a moderate number of hypermethylated loci. Compared to *SPOP* and *FOXA1* mutant tumors, two-thirds of *ERG* fusion-positive tumors had relatively low hypermethylated loci. However, one-third of *ERG* fusion-positive tumors had distinct hypermethylation patterns, which include more than twice the number of hypermethylated loci than the remaining two-thirds of the *ERG* fusion-positive tumors. DNA methylation patterns of *ETV1* and *ETV4* fusion-positive tumors were distinct from *ERG* fusion-positive tumors while *FLI1* fusion-positive tumors exhibited similar hypermethylation pattern as the latter two-thirds of the *ERG* fusion-positive tumors, having moderate hypermethylated loci.

Over 150 epigenetically silenced genes in prostate tumors were also identified by TCGA. For example, *SHF, FAXDC2, GSTP1, ZNF154*, and *KLF8* genes had hypermethylated promoters and silenced gene expression across the prostate tumor samples compared to normal prostate samples. In *ETS* fusion-positive tumors, *STAT6* was found to be epigenetically silenced, whereas this silencing was not found in prostate tumors with mutations in *SPOP* and *IDH1*. Unlike other tumor types, *SPOP* mutant tumors had epigenetically silenced *HEXA* (47).

### 3.3 Differentially methylated regions detected between AA and EA prostate cancer patients

To characterize epigenomic alterations in prostate tumors from AA patients, differential DNA methylation analyses between benign and malignant prostate tissues obtained from AA patients have been performed by multiple research groups. We found seven studies (18–24), of which six investigated DNA methylation signatures at specific genes, and two performed global epigenome analysis (**Table 2**).

For example, Barry et al. investigated DNA methylation levels at the *MYC* locus (6 CpG sites from exon 3 to the 3′ UTR) in AA and EA prostate cancer patients using pyrosequencing (18). They determined that AA patient samples were relatively hypomethylated at exon 3 of the *MYC* gene, a site that is associated with a higher Gleason score, and therefore severity of disease (18). They showed that DNA methylation level at one of the examined CpG sites is more strongly associated with Gleason score in prostate tumors from AA patients than EA patients. However, subsequent RNA-sequencing data analysis indicated that *MYC* expression was not significantly different regardless of the DNA methylation status at MYC region; the study suggested ncRNA expression to be responsible for the difference (18).

Tang et al. reported that hypermethylation of *RARB* (aka *RARß2*) was significantly associated with a higher risk of prostate cancer in AA men but not in EA men, but this study was not included since it only evaluated cancer risk according to methylation within benign prostate tissue (126). Woodson et al. studied DNA methylation of a set of genes (*GSTP1*, *RASSF1A*, *RARB*, *CD44*, *EDNRB*, *CDH1*, *ANXA2*, and *CAV1*) that were previously implicated in prostate tumorigenesis in AA patient samples and reported that *GSTP1, RASSF1A*, and *RARB* were hypermethylated and *CDH1, EDNRB*, and *CD44* were hypomethylated in tumor samples (24). This group compared the data to EA patients and found no significant differences in overall DNA methylation status for all aforementioned genes, but found *CD44* hypermethylation twice as prevalent in AA men (24). Another study by Kwabi-Addo et al. selected epigenetically altered genes in prostate cancer and found *AR, GSTP1*, *RARB, SPARC, TIMP3*, and *NKX2-5* to be hypermethylated in AA patients (21). Although *TIMP3* was identified by Lam et al. to be hypermethylated in prostate cancer, it was determined to have no prognostic utility. Two of the aforementioned genes, *SPARC* and *NKX2-5*, have been shown to be hypermethylated in prostate cancer (110, 112). When comparing these results with EA samples, Kwabi-Abbo et al. found that there was statistically significant differential methylation solely in the *TIMP3* and *NKX2-5* genes (21).

*GSTP1* was also found to be hypermethylated in AA patients by Sharad et al. (23). However, it was not found to be differentially methylated between AA and EA patients. Another study, which investigated the DNA methylation of *GSTP1* across different ethnic groups, also found it universally hypermethylated in prostate cancer, but not significantly different in DNA methylation levels between ethnic groups (20). Sharad et al. also found that the *PMEPA1* gene was found to be significantly more hypermethylated in EA prostate cancer patients compared to AA prostate cancer patients (23). This gene encodes an androgen-sensitive ligase binding protein that is now known to maintain AR protein levels in prostate tissues (23).

Unlike the aforementioned studies that characterized the DNA methylation levels of specific genes of interest, Devaney et al. performed Illumina 450K methylation arrays in AA (7 normal and 3 cancer) and EA (8 normal and 3 cancer) prostate tumor tissues (19). They identified 25 promoter-associated CpG sites that were differentially methylated by race. The most significantly differentially methylated genes were *MST1R*, *ABCG5*, and *SNRPN* (19). It is important to note that this study had a very small sample size, which may limit statistical power in identifying statistically significantly differentially DNA methylation sites from the array.

Rubicz et al. also performed Illumina HM450 methylation arrays in AA patients, subsequently with RNA-seq (22). They compared DNA methylation levels with EA from Fred Hutch Cancer Research Center (FHCRC) prostate cancer studies (127, 128). They found hypermethylation of *STOX7*, *SNRPN*, *TIMP3*, and *PMEPA1* in AA patients, which were subsequently found to be differentially methylated between AA and EA (22). The hypermethylation of *TIMP3* and *PMEPA1* were found by several other studies (20, 21, 23, 24, 126). However, *SNRPN* hypermethylation did not correspond to lower expression in this study, unlike the findings by Devaney et al. (19). Additionally, they were able to identify differentially methylated regions between AA prostate cancer patients with and without prostate cancer recurrence in the gene bodies and promoter regions of genes involved in specific tumorigenic biological processes such as protein kinase activity and metal ion binding activity. For example, *CDKL2*, *FOXA2*, *NEUROG1*, and *GCK* were found to be hypermethylated at promoter regions with corresponding decreased transcription levels in AA prostate cancer patients with recurrence compared to patients without recurrence (22).

The lack of consistency in the findings between studies could be attributed to insufficient sample sizes, differences of profiling methods, the heterogeneous nature of the subpopulation itself, or non-biological factors. Overall, this shows the necessity for further whole genome methylation sequencing analysis of AA patient prostate cancer tissue samples.

## 4 Transcriptomic alterations linked to African American prostate cancer patients

### 4.1 Methods to profile gene expression in cancer

Genomic and epigenomic alterations in tumors lead to the reprogramming of gene expression patterns. Profiling gene expression is crucial to understand carcinogenesis and identify therapeutic targets. For example, amplification of the *MYC* oncogene leads to its overexpression, which in turn activates its target genes, promoting cell proliferation. Moreover, some tumor cells can be targeted directly based on the overexpression of certain transmembrane proteins. For instance, prostate specific membrane antigen (PSMA), which is upregulated in many metastatic prostate tumors, can be targeted with its radiolabeled inhibitor and used for imaging modalities or radiotherapy (129, 130). By comparing gene expression patterns, transcriptional networks and signaling pathways altered in tumors can be also revealed.

In order to better develop the global gene expression profiles of cancer cells, researchers have utilized mRNA quantification techniques such as Reverse Transcriptase Quantitative Polymerase Chain Reaction (RT-qPCR), RNA microarrays, and RNA-sequencing (131, 132). All techniques utilize the conversion of RNA transcripts into cDNA through reverse transcription, but RT-qPCR is limited due to its probing of only known gene regions. Both RNA microarrays and RNA-sequencing are instead capable of profiling entire transcriptomes. However, these two platforms differ in their benefits and limitations. Microarrays allow for the expression profiling of thousands of transcripts through cDNA hybridization using probes simultaneously. This methodology can only interrogate expression of known transcripts and selected exons. It also has a lack of specificity, or high background noise due to cross-hybridization of multiple transcripts to the same probe (132, 133). Examples of microarrays commonly used include the GeneChip Human 525 Exon 1.0 ST, which can interrogate over 1 million exon clusters, with four probes per exon on average. Another commonly used microarray with a smaller breadth of capabilities is the GeneChip HG-U133A 2.0, which interrogates 14,500 well-known genes.

RNA-sequencing instead uses NGS to fully catalog the transcriptome of a sample through sequencing of cDNA transcripts, regardless of whether they are known transcripts or not (134). RNA-sequencing allows for the characterization of transcriptomic features such as alternative splicing events and antisense transcripts (134). The most popular RNA-sequencing technique involves the capture of mRNA based on the presence of polyA tails at the 3’ end (polyA RNA-seq); an example of this technique is the Illumina TruSeq RNA library preparation assay (135). Another type of RNA-sequencing (total RNA-seq) is to capture the total RNA after depleting ribosomal RNA, which profiles RNA transcript levels of coding genes, noncoding regions, and small RNAs (136). The major limitation of this method is that it is more expensive than microarray technology. Additionally, RNA-seq libraries are relatively difficult to prepare and analyze compared to RNA microarrays. Overall, when studying global transcriptomic features of tumor cells and tissues, RNA microarrays allow for the consistent generation of tumor expression profiles, and RNA-sequencing technology allows for the discovery of novel transcriptomic events and the quantification of their overall expression.

### 4.2 Genes and signaling pathways dysregulated in prostate cancer

RNA-sequencing and microarray technologies have been used to analyze the transcriptomes of prostate carcinomas to better understand the gene expression patterns that are specific to the disease and contribute to tumorigenesis. Many of these expression changes are consequences of genomic or epigenomic alterations, which are used to categorize the molecular subtypes mentioned above. Gene expression changes found in prostate cancer can be understood through their effects on AR signaling (137). AR is essential for the normal growth and development of the prostate gland. Individuals with defective AR signaling do not experience prostate enlargement, and inhibiting AR activity results in reduced prostate size and symptoms (137). During prostate tumor cell transformation and carcinogenesis, AR activity is dysregulated, and androgen dependence and sensitivity is altered. For example, when AR transcriptional activity has been characterized in primary prostate tumors by the expression pattern of the *AR* gene itself as well as the 20 previously described AR target genes, tumors with *SPOP* or *FOXA1* mutations have the highest AR transcriptional activity (47). This pattern is supported by the fact that SPOP mutant has been shown to induce AR signaling (138). FOXA1 mutations in prostate cancer cells are reported to reduce the number of AR binding sites but replaces AR function by increasing the activities of AR target genes (139) AR coactivators and FOXA1 are known to interact with AR by binding to numerous enhancers to increase AR signaling (77). However, AR activity varies among tumors with fusion involving an AR-controlled *ETS* gene (i.e. *TMPRSS2-ERG*, *TMPRSS2-ETV1*, *TMPRSS2-ETV4*, *TMPRSS2-FLI1* fusions) (47). *TMPRSS2-ETS* fusion gene is reported to be regulated by AR, but it is highly expressed in both primary and metastatic prostate tumors, independent of *AR* transcription levels (47). Mechanistic models have been proposed regarding *AR* transcription and signaling, but ultimately the exact mechanisms that result in the formation of molecular subtypes throughout tumorigenesis is unknown (134).

AR signaling alterations are found to be associated with metastatic prostate tumors. For example, the expression of AR-V7 transcript variant that lacks LBD in metastatic prostate tumors is associated with resistance to hormone therapy (140). AR-V7 expression was not associated with the molecular subtypes of primary prostate tumors and AR transcriptional activity, however (47). *AR* expression in metastatic prostate cancer is also reported to altered by epigenetic changes such as enhancer and chromatin interaction changes (141). Additionally, *AR* expression is affected by changes in ncRNA transcripts, and subsequently affects the expression of other ncRNA transcripts (69).

There are additional genes and signaling pathways reported to be altered in prostate cancer. For example, prostate tumor samples with *SPOP* mutations have been associated with the overexpression of *SPINK1* through activating the mitogen-activated protein kinase (MAPK) pathway (142). Other significant pathways found to be upregulated in prostate tumors are the phosphatidylinositol-3-kinase (PI3K)/Akt/mammalian target of rapamycin (mTOR), Ras/MAPK, DNA repair, and receptor tyrosine kinase pathways (143, 144).

### 4.3 Genes differentially expressed between AA and EA prostate cancer patients

Characterizing the genes and pathways linked to AA prostate tumor samples will allow researchers to better understand differences in tumor progression and treatment response between subgroups. When we searched studies that generated RNA-sequencing or microarray data from prostate tissue samples, and directly compared gene expression profiles between AA and EA prostate cancer patients, we found eight different studies (25–32) (**Table 3**).

The largest study among the 8 studies was performed by Rayford et al., which analyzed the gene expression patterns between 596 AA and 556 EA prostate cancer patients using the Human Exon 1.0 ST microarrays, as well as TCGA RNA-seq data (30). Major findings included the significant association of *SPINK1* overexpression in AA patients relative to EA patients, and lack of *TMPRSS2-ERG* fusions (30). SPINK1 was also found to be overexpressed at the protein level more frequently in AA patient samples in the study by Khani et al. (12). The lack of *TMPRSS2-ERG* fusions in AA patients was also noted in patient samples analyzed by Echevarria et al. (25). Indeed, five (*BCL6*, *EMP1*, *MYADM*, *SRGN*, and *TIMP3*) of the six differentially expressed genes (*APOD*, *BCL6*, *EMP1*, *MYADM*, *SRGN*, and *TIMP3*) that Echevarria et al. suggested to be useful as AA-specific prostate cancer biomarkers were primarily present in the *TMPRSS2-ETS* fusion negative tumor samples (25).

Additionally, Rayford et al. found that the *CRYBB2* and *GSTM3* genes were upregulated in AA prostate cancer patients than EA patients (30). These genes were also reported to be upregulated in TCGA prostate cancer AA patients. *CRYBB2* specifically was found by two studies to be overexpressed in AA men (28, 30) while another study indicated that it was underexpressed in AA men relative to EA men prostate tissue samples (29). Rayford et al. also reported that the biological pathways upregulated in AA patients were related to inflammatory response (e.g. *IL33, IFNG, CCL4, CD3, ICOSLG*), whereas the biological pathways upregulated in EA patients were related to DNA repair (e.g. *MSH2*, *MSH6*), metabolism, cell proliferation, and cell cycle (30). Inflammatory and immune pathway dysregulation was noted by other studies as well. A study by Hardiman et al. in 2016, which performed RNA-sequencing in 10 AA and 17 EA prostate cancer patients, observed that multiple immune and inflammatory pathways (e.g. *IL2RG*, *CD1C*, *CD207*, *CCL4*, *CCL8*, *CXCR4*) were upregulated in AA patients relative to EA patients (26). A study by Rahmatpanah et al., which analyzed RNA-seq data obtained from 15 AA and 30 EA prostate tumors, observed an upregulation of inflammatory and immune pathways (e.g. *CXCL10*, *CXCL2*, *HLA-A*, *CCL2*) (29). Using the Affymetrix GeneChip HU-U133A 2.0 array, Wallace et al. observed upregulation in the inflammatory pathway (e.g. *CXCR4*, *CCL5*, *CCR7*) in tumor samples from 33 AA patients relative to 36 EA patients as well (32).

Nagaya et al. performed RNA-sequencing in prostate tumors from 31 AA and 30 EA men and found 45 differentially expressed genes (28). The identified genes were not involved in the inflammatory and immune pathway. The most notable findings in this study were that four of the 45 differentially expressed genes were found to be in the neuroactive ligand pathway: *GALR1, CHRM3, NPFFR1*, and *S1PR3* (28). *S1PR3* expression level was higher in AA than EA prostate cancer patients whereas *GALR1, CHRM3*, and *NPFFR1* expression level was lower in AA than EA prostate cancer patients (28). Expression changes found for *NPFFR1* and *S1PR3* genes were not reported by other studies (25–32). However, *GALR1* was also found to be downregulated in AA patient samples compared to EA patient samples in one other study (27) and *CHRM3* was found to be downregulated in two other studies (25, 30). Hardiman et al., which performed RNA-seq in prostate tumors from 33 AA and 27 EA men, reported downregulation of *GALR1* in AA patients relative to EA patients, and reported differential expression of other genes in the neuroactive ligand pathway (27). For example, *GABRP*, *ADORA2B*, *TACR1*, *TAAR1*, and *GABRQ* were modestly downregulated in AA compared to EA, and *AGTR1*, *F2RL2*, *NPY4R* and *GRIN3A* were upregulated in AA compared to EA (27). Rahmatpanah et al. found that *GRIN3A* was also downregulated in AA relative to EA patients (29). The involvement of aberrant neuroactive ligand signaling pathway has been reported in other cancer types (145, 146).

Another pathway found to be differentially expressed between AA and EA prostate cancer patients across studies was the PI3K-Akt pathway, which is involved in cell survival, growth, and proliferation. For example, Rahmatpanah et al. found that genes involved in this pathway such as *PIK3CA* were overexpressed in AA samples relative to EA samples (29). Hardiman et al. also reported that PI3K pathway is altered and that some genes involved in the PIK3-Akt pathway were significantly upregulated (*THBS4*, *CREB3L1*, *TNN*, *COL4A4*, *COL4A3*, *COL2A1*, *FGF12*, *MYC*, and *GNG13*) and downregulated (*SGK1*, *ANGPT2*, *FGF11*, *IL4*, *IL6*, *ANGPT4*, *THBS2*, *FLT4*, *NTRK2*, *PIK3R6*, *LAMA5*, and *MET*) between AA and EA (27). However, these genes were not found to be differentially regulated in the Rahmatpanah study (29).

The Ras/MAPK pathway has also been implicated in prostate cancer. One study by Timofeeva et al. showed the overexpression of *SOS1*, an activator of this pathway, in prostate cancer tissues compared to normal prostate tissues at both mRNA and protein levels (31). The researchers showed that *SOS1* expression was two-fold higher in AA men relative to EA men with prostate cancer (31). Interestingly, its overexpression was correlated to higher Gleason score, indicating that *SOS1* overexpression could be a biomarker for AA disease severity (31).

Although the above eight studies revealed genes that are differentially expressed between AA and EA, these data have not entirely elucidated differences in prostate tumorigenesis in AA versus EA patients. Wallace et al., for example, compared their differentially expressed genes between AA and EA to the top 80 differentially expressed genes in prostate cancer (tumor vs. normal) and found no overlap (32). It is possible, however, that the differentially expressed genes classically found in prostate cancer were not studied with a large amount of AA patient samples, or that there are relatively less significant race-specific biological factors contributing to the progression of disease.

## 5 Discussion

In this study, we reviewed more than 20 studies that analyzed the genomes, epigenomes, and transcriptomes of prostate tumor tissue samples from AA and EA patients. Although there were several AA-specific alterations reported in more than one study, many of the findings reported by multiple research groups were contradictory to others. Another recent review on prostate tumor genomics also reported that there was no clear association between specific genetic changes and race (147). This could be due to the large variance in sample sizes of both EA and AA groups as the size of samples is crucial for getting statistically significant findings. To further evaluate the biological contributions, if any, to the clinical disparities that disproportionately affect AA men with prostate cancer, there needs to be a greater number of AA-specific studies that take account of genome-wide genomic, epigenomic, and transcriptomic approaches. For example, although there were a significant number of genes that are differentially expressed between normal prostate and prostate cancer, those genes were characterized in patient cohorts that were largely composed of EA men. Additionally, there are very few epigenetic studies relative to the number of genetic and transcriptomic studies. Evaluating prostate cancer in AA men with a whole genome approach with equally large sample sizes could possibly allow researchers to elucidate AA-specific biomarkers. It is recently reported that the RESPOND study (Research on Prostate Cancer in Men of African Ancestry: Defining the Roles of Genetics, Tumor Markers, and Social Stress) will characterize molecular genetic signatures from 10,000 AA prostate cancer patients.

Additionally, the methods by which the prostate tissue samples are obtained can bias study results. Prostate tissue for these studies was obtained from patients by different kinds of methods (e.g. biopsy versus prostatectomy, fresh versus frozen versus formalin fixed paraffin embedded tissue (FFPE), etc), which may lead to results that are not comparable. For example, the treatment of tissue samples *ex vivo* (e.g. FFPE) may affect tissue conditions. Moreover, the prostate tumor tissue samples themselves can be heterogeneous within the given subpopulation. For instance, tumor stage, cellularity, and microenvironment can affect genomic, epigenomic, and transcriptomic signals. In addition, there is substantial molecular heterogeneity among prostate tumors. Distinct genetic and molecular signatures have found to define molecular subgroups of prostate cancer. Considering the greater relative incidence and mortality that is suffered by AA men with prostate cancer, it is important that we further characterize prostate cancer molecular subgroups with appropriate AA patient representation.

Lastly, most studies rely on ethnicity information inferred from self-reported ancestry. It has been recently shown by Schumacher et al. that, upon analyzing the GENIE 8.0 registry, genetic mutational frequency differences determined across patients of varying self-reported race were either insignificant or in non-clinically actionable regions as of present, but this study was not included in our analysis due to unclear cohort sizes (148). It is important to confirm and measure ethnicity information from samples using genetic ancestry informative marker data that quantify the high heterogeneity among subpopulations.

In conclusion, to better understand racial disparities in prostate cancer, molecular genetic signatures that are differentially associated between AA and EA patients were characterized using various techniques. By reviewing over 20 published studies, we revealed that heterogeneous genomic, epigenomic, and transcriptomic alterations are found between AA and EA prostate cancer patients. However, as results are controversial across different studies, additional large-scale investigations that take into account of potential confounding factors are greatly needed. Elucidating molecular mechanisms of tumorigenesis associated with tumor subgroups will provide valuable resources to identify novel biomarkers and treatment modalities to improve the disparity of clinical outcomes between AA and EA patients.

## Author contributions

CS and SKR wrote the initial draft and finalized the manuscript. CS, AH, and SKR collected the data and prepared the figure and tables. SKR conceptualized and supervised the manuscript. All authors contributed to the article and approved the submitted version. 
